# Neutralizing Antibodies and Cellular Immune Responses Against SARS-CoV-2 Sustained One and a Half Years After Natural Infection

**DOI:** 10.3389/fmicb.2021.803031

**Published:** 2022-03-03

**Authors:** Li-na Yan, Pan-pan Liu, Xu-gui Li, Shi-jing Zhou, Hao Li, Zhi-yin Wang, Feng Shen, Bi-chao Lu, Yu Long, Xiao Xiao, Zhen-dong Wang, Dan Li, Hui-ju Han, Hao Yu, Shu-han Zhou, Wen-liang Lv, Xue-jie Yu

**Affiliations:** ^1^State Key Laboratory of Virology, School of Public Health, Wuhan University, Wuhan, China; ^2^The Department of Clinical Laboratory Medicine, Hubei 672 Orthopaedics Hospital, Wuhan, China; ^3^The First School of Clinical Medicine, Hubei University of Chinese Medicine, Wuhan, China; ^4^Department of Clinical Laboratory Medicine, Hubei University of Chinese Medicine Huangjiahu Hospital, Wuhan, China; ^5^College of Acupuncture and Orthopedics, Hubei University of Chinese Medicine, Wuhan, China; ^6^Clinical College, Hubei University of Chinese Medicine, Wuhan, China; ^7^School of Public Health, Xi’an Medical University, Xi’an, China; ^8^Department of Neuroscience, Cell Biology, and Anatomy, University of Texas Medical Branch, Galveston, TX, United States

**Keywords:** neutralizing antibody, T-cell response, antigen-specific, SARS-CoV-2, COVID-19

## Abstract

**Background:**

COVID-19 has caused more than 2.6 billion infections and several million deaths since its outbreak 2 years ago. We know very little about the long-term cellular immune responses and the kinetics of neutralizing antibodies (NAbs) to SARS-CoV-2 because it has emerged only recently in the human population.

**Methods:**

We collected blood samples from individuals who were from the first wave of the COVID-19 epidemic in Wuhan between December 30, 2019, and February 24, 2020. We analyzed NAbs to SARS-CoV-2 using pseudoviruses and IgG antibodies to SARS-CoV-2 spike (S) and nucleocapsid (N) protein using enzyme-linked immunosorbent assay in patients’ sera and determined SARS-CoV-2-specific T-cell responses of patients with ELISpot assays.

**Results:**

We found that 91.9% (57/62) and 88.9% (40/45) of COVID-19 patients had NAbs against SARS-CoV-2 in a year (10–11 months) and one and a half years (17–18 months), respectively, after the onset of illness, indicating that NAbs against SARS-CoV-2 waned slowly and possibly persisted over a long period time. Over 80% of patients had IgG antibodies to SARS-CoV-2 S and N protein one and a half years after illness onset. Most patients also had robust memory T-cell responses against SARS-CoV-2 one and a half years after the illness. Among the patients, 95.6% (43/45) had an IFN-γ-secreting T-cell response and 93.8% (15/16) had an IL-2-secreting T-cell response. The T-cell responses to SARS-CoV-2 were positively correlated with antibodies (including neutralizing antibodies and IgG antibodies to S and N protein) in COVID-19 patients. Eighty percent (4/5) of neutralizing antibody-negative patients also had SARS-CoV-2-specific T-cell response. After long-term infection, protective immunity was independent of disease severity, sex, and age.

**Conclusions:**

We concluded that SARS-CoV-2 infection elicited a robust and persistent neutralizing antibody and memory T-cell response in COVID-19 patients, indicating that these sustained immune responses, among most SARS-CoV-2-infected people, may play a crucial role in protection against reinfection.

## Introduction

Coronavirus disease 2019 (COVID-19), caused by a newly discovered beta coronavirus, severe acute respiratory syndrome coronavirus 2 (SARS-CoV-2), has become a global pandemic with no end in sight. As of December 12, 2021, 267.9 million cases of COVID-19 had been confirmed worldwide and 5.2 million cases had died (World Health Organization [WHO], 2021). The protective immune responses triggered by viral infection consist of two main components, humoral and cell-mediated immune responses. Neutralizing antibodies (NAbs) can neutralize the viral pathogen and are considered crucial for protection against viral infections. Specific T-cell immune responses are usually directed against virus-infected cells, accelerate viral clearance, and restrict viral spread *in vivo* (Feng et al., 2021). Studies have indicated a protective role of NAbs and antigen-specific memory T-cell responses in COVID-19 patients (Addetia et al., 2020; Huang et al., 2020). SARS-CoV-2 convalescent patients usually generate robust immune responses in the early stages of convalescence (Choe et al., 2020; Robbiani et al., 2020; Rydyznski Moderbacher et al., 2020; Wang Y. et al., 2020; Cassaniti et al., 2021). Previous studies reported that SARS-CoV-2-specific T-cell responses occurred in virtually all patients (Grifoni et al., 2020; Feng et al., 2021; Sandberg et al., 2021). However, the long-term role of NAbs and cellular immunity against SARS-CoV-2 is unknown due to the recent outbreak of COVID-19, which may contribute to protection against reinfection.

In this study, blood samples were obtained from COVID-19 patients with mild and severe illness from 1 to 18 months after disease onset to evaluate neutralizing antibodies, antigen-specific antibodies, and T-cell responses to SARS-CoV-2.

## Materials and Methods

### Blood Samples

**FIGURE 1:**
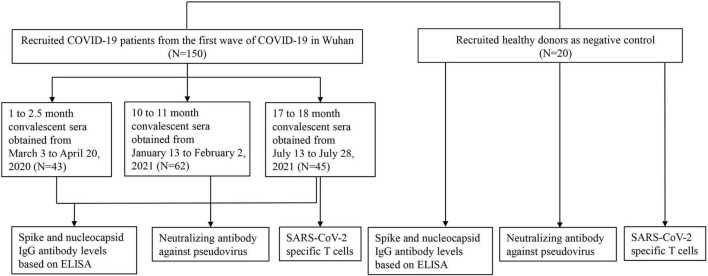
Enrollment of COVID-19 patients and healthy persons. A total of 150 COVID-19 patients including inpatients and convalescent patients were enrolled in this study and patients’ sera were obtained from 1 to 18 months after the onset of illness in three periods (1–2.5, 10–11, and 17–18 months). A total of 20 healthy persons were recruited as the negative control.

The study was conducted with the approval of the Ethics Committee of Wuhan University (2020YF0051). Informed consent was obtained from all participants. A total of 170 participants were recruited in this study. Inclusion criteria were as follows: (1) no history of COVID-19 vaccination; and (2) no individuals living with human immunodeficiency virus (HIV). All COVID-19 cases were diagnosed according to the guidelines for the diagnosis and treatment of new coronavirus pneumonia issued by the Chinese government, and were confirmed to be infected with SARS-CoV-2 by RT-PCR test of nasopharyngeal swabs. A total of 150 hospitalized patients who were the first wave of COVID-19 in Wuhan between December 30, 2019, and February 24, 2020 were recruited (Figure 1). The 1- to 2.5-month sera were obtained from inpatients, and the 10- to 11-month sera and the 17- to 18-month sera were obtained from volunteers who had been hospitalized and recovered from COVID-19. We divided the observation period into 1–2.5 months (*n* = 43), 10–11 months (*n* = 62), and 17–18 months (*n* = 45). The samples collected at different time points came from different patients. A total of 20 healthy control subjects who were neither infected with SARS-CoV-2 nor vaccinated against COVID-19 were recruited. All sera were aliquoted and stored at −80°C.

### Generation of the Human Angiotensin-Converting Enzyme 2 (ACE2) Overexpression Cell Lines (Vero-E6_*ACE*2_ Cells)

The lentiviral expression vectors were used to transfect Vero-E6 cells to stably express human ACE2. The retroviral plasmid for human ACE2 expression (pCDH-CMV-hACE-EF1-RFP) was purchased from Tsingke Biological Technology (Nanjing, China). HEK293T cells were transfected with the retroviral vector pCDH-CMV-hACE-EF1-RFP and the packaging plasmids pMD2.G and psPAX2 at a ratio of 2:1:1 with Lipofectamine 3000 (Invitrogen, Carlsbad, CA, United States). After 48 h of incubation, the supernatant was harvested and the lentiviruses in the supernatant were purified with polyethylene glycol (PEG) 8000 (Solarbio, Beijing, China). Confluence monolayers (80–90%) of Vero-E6 cells were infected with purified lentiviruses with polybrene (2 μg/ml). Stable Vero-E6_*ACE*2_ cells were selected by puromycin resistance.

### Production of SARS-CoV2 S Pseudovirus

To construct an HIV-1 pseudovirus carrying the spike (S) protein of SARS-CoV-2, the full length of the S gene with a C-terminal 19-amino-acid deletion from the Wuhan-Hu-1 strain (GenBank: MN908947) was codon-optimized and synthesized (Sino Biology Inc., Shanghai, China). The S gene was cloned into the eukaryotic expression plasmid pCMV to generate the plasmid pCMV-3Flag-SARS-CoV-2-S19del. The HIV-1 NL4-3 ΔEnv Vpr luciferase reporter vector (pNL4.3-R-E-luc) was kindly provided by Professor Cheguo Cai (Wuhan University, Wuhan, China). HEK293T cells were co-transfected with pNL4.3-R-E-luc and pCMV-3Flag-SARS-CoV-2-S19del using Lipofectamine 3000 to package the pseudovirus. The Supernatant containing pseudovirus was collected at 72 h post-transfection. To construct the vesicular stomatitis virus (VSV) pseudovirus as control, plasmid pMD2.G was co-transfected with plasmid pNL4.3-R-E-luc.

Pseudovirus titers were determined on Vero-E6_*ACE*2_ cells by measuring relative luminescence units (RLU). Briefly, Vero-E6_*ACE*2_ cells (2 × 10^4^ cells/well) were seeded into 96-well culture plates and infected with 50 μl of pseudovirus with polybrene (2 μg/ml). After incubation for 24 h, the supernatant containing pseudovirus was removed and replaced with fresh Dulbecco’s modified Eagle’s medium containing 2% fetal bovine serum (Gibco, Rockville, MD, United States). After 72 h post-infection, RLU was detected using the Bio-Lite Luciferase Assay kit (Vazyme, Nanjing, China). The luminescence was read using a Tecan Spark multifunction microplate reader (Tecan, Austria).

### Confirm the Expression of SARS-CoV-2 Pseudovirus by Western Blot

To confirm the expression of SARS-CoV-2 pseudovirus, HEK293T cells were cotransfected with pNL4.3-R-E-luc and pCMV-3Flag-SARS-CoV2-S19del using Lipofectamine 3000, and the virus-containing supernatant and cell lysates were collected separately. To construct the VSV pseudovirus as control, plasmid pMD2.G was cotransfected with pNL4.3-R-E-luc. Twelve milliliters of SARS-CoV-2 or VSV pseudoviruses was concentrated with PEG 8000 and resuspended with 20 μl of 5 × sodium dodecyl sulfate-polyacrylamide gel electrophoresis (SDS-PAGE) loading buffer. Cell lysates were denatured in a 5 × SDS-PAGE loading buffer. Proteins were electrophoresed on a 10% SDS-PAGE and transferred onto a polyvinylidene difluoride membrane for 2.5 h at 200 mA at 4°C. Immunoblots were probed with primary antibodies (SARS-CoV-2 convalescent patient serum, anti-Flag, and anti-β-actin antibodies, Proteintech, Wuhan, China) and secondary antibodies conjugated with horseradish peroxidase. Protein bands were detected using Western Lightning Plus ECL (PerkinElmer, Boston, MA, United States).

### Neutralization Assay Based on the Pseudovirus

Vero-E6_*ACE*2_ cells (2 × 10^4^ cells/well) were seeded in 96-well plates. For the pseudovirus neutralization assay, heat-inactivated serum samples were diluted in twofold increments with Dulbecco’s modified Eagle’s medium containing 2% fetal bovine serum. SARS-CoV2-S pseudoviruses (50 μl, approximately 5 × 10^4^ RLU) were co-incubated with 50 μl of diluted sera of COVID-19 patients for 1 h at 37°C and then the sera-pseudoviruses mixture was added to 96-well Vero-E6_*ACE*2_ cells. Sera of healthy donors were used as negative serum controls. VSV pseudovirus was a negative virus control to prove the specificity of the neutralization assay-based pseudovirus. Fresh medium was added to each well 24 h post-infection and RLU was measured 72 h post-infection. The percentage of neutralization was calculated as (RLUvirus − RLUvirus + serum)/(RLUvirus − RLUmock) × 100. The 50% neutralization (ND_50_) was expressed as the reciprocal dilution of serum, which resulted in a 50% reduction in RLU compared to the positive virus control. In this study, neutralizing antibody titers were calculated as ND_50_. The ND_50_ titer of ≥1:8 indicated positivity. All serum samples were tested at least in duplicate.

### Enzyme-Linked Immunosorbent Assay

We detected the SARS-CoV-2 IgG antibody to S and nucleocapsid (N) in 1- to 2.5-month sera, 10- to 11-month sera, and 17- to 18-month sera using ELISA. The IgG levels in sera were tested using the commercially available SARS-CoV-2 human S antibody Titer Assay Kit and SARS-CoV-2 human N antibody Titer Assay Kit (Nanjing Miaodi Biotechnology Co., Ltd., Nanjing, China) (Liu et al., 2021). A total of 100 μl of diluted serum sample (1:50 for S and 1:100 for N) was added to each well coated with purified SARS-CoV-2 S or N protein. Following 30 min of incubation at 37°C, each well was washed and was incubated with horseradish peroxidase-labeled anti-human IgG. Tetramethylbenzidine and H_2_O_2_ substrate were used to develop color. After color development stopped, the optical density (OD) values were measured at 450 nm (OD_450_) with a microplate reader. Positive control and negative control were performed as the same procedure. Based on the manufacturer’s instructions, samples with OD_450_ greater than the cutoff value were considered positive.

### SARS-CoV-2 Peptides for the T-Cell Enzyme-Linked Immunospot Assay (ELISpot)

For the T-cell ELISpot assay, the SARS-CoV-2 S peptide pools (Sino Biological, Beijing, China) containing S1 and S2 peptide pools were used, which mainly consisted of 15-mer sequences with 11-amino-acid overlap. The S1 peptide pool consists of 169 peptides (amino acids 1–686) and the S2 peptide pool consists of 144 peptides (amino acids 687–1,273), with a purity of >95% as determined by SEC-HPLC analysis.

### ELISpot Assay

To investigate SARS-CoV-2-specific T-cell responses in convalescent patients, we used the human IFN-γ and IL-2 ELISpot Kits (Dakewe Biotech, Shenzhen, China). Fresh peripheral blood mononuclear cells (PBMCs) were isolated from the blood of convalescent COVID-19 patients 17–18 months after disease onset. PBMCs from healthy individuals who had not been vaccinated against COVID-19 served as controls. As a standard, 5 × 10^5^ PBMCs per well were stimulated with the S1 and S2 mixture peptides at a concentration of 1 μg/ml of each peptide for 24 h. An equimolar amount of dimethyl sulfoxide (DMSO) and phytohemagglutinin (PHA, Dakewe, 2.5 μg/ml) were used as negative and positive controls, respectively. Spots were counted using an ELISpot reader (Mabtech, Stockholm, Sweden). The threshold for a positive reaction was set at three times above the mean value of the spots of the negative controls.

### Statistical Analysis

Categorical variables were described as percentages, and compared using the Chi-squared test. Continuous variables were described using geometric mean antibody titers (GMT), median, and interquartile range (IQR). The Mann–Whitney *U* test was used to compare differences between groups. Spearman rank correlation test was used to evaluate the correlation between the levels of different antibodies and the correlation between antibody levels and T-cell responses. The generalized linear model (GLM) was applied to evaluate the association between immune response (including antibodies and T-cell responses) and potential factors (i.e., disease severity, sex, and age). All analyses were performed using SPSS 22.0 software (SPSS Inc., Chicago, IL, United States). Graphs were generated using GraphPad Prism 8.3.0. *p* < 0.05 was considered statistically significant. *p < 0.05, ^**^p < 0.01, ^***^p < 0.001.

## Results

### Characteristics of the Patients

In this study, we recruited 150 COVID-19 patients and 20 healthy donors (Figure 1). Among the 150 COVID-19 patients who participated in this study, 43 sera were obtained in 1–2.5 months, 62 sera were obtained in 10–11 months, and 45 sera were obtained in 17–18 months after the onset of illness. Patients were grouped according to the severity of the disease. Patients with fever, fatigue, runny nose, cough, myalgia, and mild pneumonia on imaging examination were included in the mild group; patients with dyspnea, hypoxemia, acute respiratory distress syndrome, septic shocks, and multifunctional organ failure requiring intensive care were included in the severe group. General information about patients enrolled in neutralizing antibody tests is listed in Table 1, including sex, age, and the severity of COVID-19. The median age of the patients was 55 years (IQR, 47–63 years) and 68 (45.6%) were female. Among them, 81 (54.4%) were younger than 60 and 36 (39.7%) were over 60 years old. Eighty-four (56.4%) patients had mild clinical manifestations and 65 (43.6%) were in severe condition.

**TABLE 1:** General information about cases enrolled in the neutralizing antibody test.

Characteristic	Total (*N* = 170)	COVID-19 patients			Healthy persons (*N* = 20)
		1–2.5 months (*N* = 43)	10–11 months (*N* = 62)	17–18 moths (*N* = 45)	

Admission/discharge		Inpatients	Convalescents	Convalescents	NA
**Sex, *n* (%)**					
Male	75 (44.1)	22 (51.2)	21 (29.4)	25 (55.6)	7 (35.0)
Female	95 (55.9)	21 (48.8)	41 (70.6)	20 (44.5)	13 (65.0)
Age, years, median (IQR)	53 (42.5, 63)	59.5 (36.5, 70)	55.0 (30, 86)	55.4 (33, 85)	38 (27, 42)
**Age (year)**					
<60, *n* (%)	110 (64.7)	22 (51.2)	42 (67.7)	28 (62.2)	18 (90.0)
≥60, *n* (%)	60 (35.3)	21 (48.8)	20 (32.3)	17 (37.8)	2 (10.0)
**Severity of COVID-19, *n* (%)**					
Mild	85 (56.7)	15 (34.9)	34 (54.8)	36 (80.0)	NA
Severe	65 (43.3)	28 (65.1)	28 (45.2)	9 (20.0)	
The median time between symptom onset to blood sampling (min, max)		50 (21,76) days	10–11 months	17–18 months	NA

*IQR, interquartile range; NA, not available.*

### Neutralization Assay With SARS-CoV-2 and Vesicular Stomatitis Virus Pseudoviruses

**FIGURE 2:**
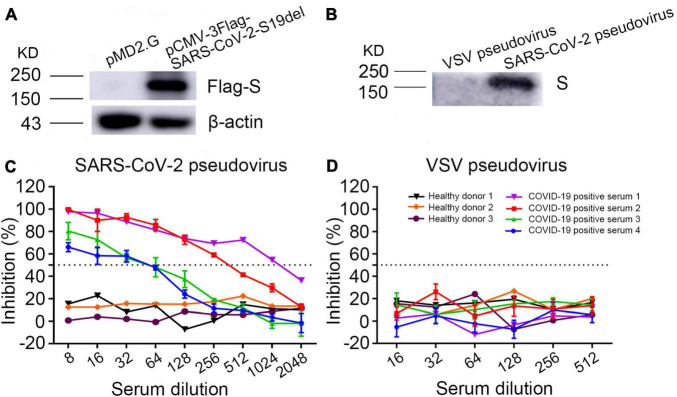
Validation of the SARS-CoV-2 pseudovirus neutralizing assay. **(A)** Detection of SARS-CoV-2 spike protein expression in plasmid pCMV-3Flag-SARS-CoV-2-S19del transfected cell lysates by Western blot using anti-Flag antibody. Plasmid pMD2.G was used as the negative control. **(B)** Detection of SARS-CoV-2 spike protein expression in PEG 8000 concentrated pseudoviruses from cell culture supernatant with a serum of COVID-19 convalescent patient. VSV pseudovirus was used as the negative control. **(C,D)** Inhibition of SARS-CoV-2 pseudovirus or VSV pseudovirus using COVID-19 patients’ and healthy persons’ sera.

Western blot showed that anti-Flag antibody detected a 190-kDa band in the cell lysates of SARS-CoV-2 S plasmid pCMV-3Flag-SARS-CoV-2-S19del transfected cells, but not in the plasmid pMD2.G transfected cells (Figure 2A), indicating that SARS-CoV-2 S protein was expressed in the S plasmid transfected cells. SARS-CoV-2 pseudoviruses were purified from cell culture supernatant using PEG 8000. Western blot showed that a COVID-19 patient serum reacted with a 190-kDa band in the purified SARS-CoV-2 pseudovirus, but not in the VSV pseudovirus (Figure 2B), indicating that SARS-CoV-2 pseudovirus S protein was correctly expressed in the cell culture supernatant.

Determination of neutralization antibody titers in sera depended on measuring the inhibition rate of RLU from pseudovirus in cells, which corresponded to the inhibition of virus entry into Vero-E6_*ACE*2_ cells. Dose-dependent inhibition of SARS-CoV-2 pseudovirus was observed in seropositive serum samples; in contrast, the sera of healthy donors showed no significant changes in the RLU at any dilution (Figure 2C). As expected, sera of both COVID-19 patients and healthy donors had no inhibition against VSV pseudovirus (Figure 2D). These results demonstrated that COVID-19 positive sera specifically neutralized the SARS-CoV-2 pseudovirus and the SARS-CoV-2 pseudovirus can substitute live SARS-CoV-2 to test neutralizing antibodies.

### Neutralizing Antibody Kinetic Change

**FIGURE 3:**
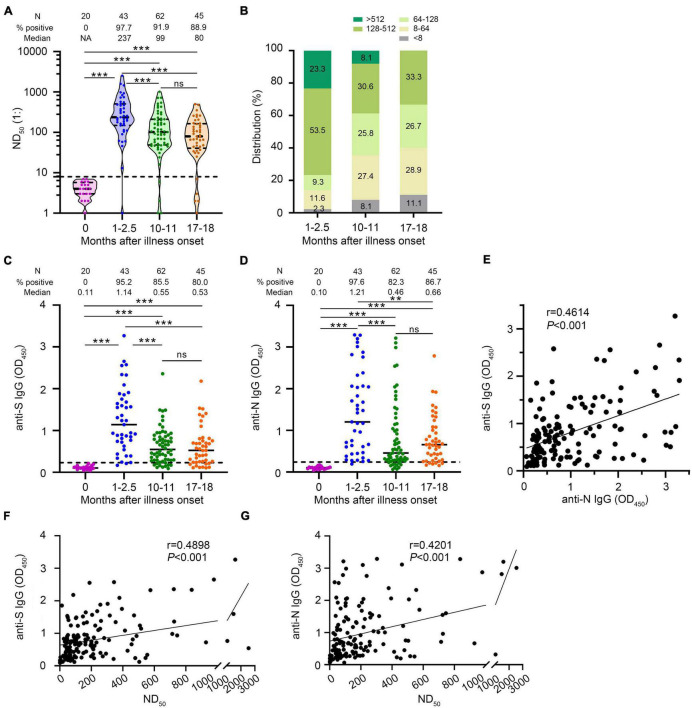
Changes in antibody levels against SARS-CoV-2 after illness onset. **(A)** Neutralizing antibody titers (ND_50_) of COVID-19 patients’ serum sample. **(B)** Distribution of the neutralizing antibody titer in serum samples from COVID-19 patients. **(C)** SARS-CoV-2 spike (S) IgG antibody levels. **(D)** SARS-CoV-2 nucleocapsid (N) IgG antibody levels. **(E)** Correlation of anti-S IgG levels and anti-N IgG levels (*N* = 150). **(F)** Correlation of ND_50_ and anti-S IgG levels (*N* = 150). **(G)** Correlation of ND_50_ and anti-N IgG levels (*N* = 150). Statistical significance of antibody levels was determined using the Mann–Whitney *U*-test. A two-sided Spearman rank correlation test was used to evaluate the correlation of the levels of different antibodies. Healthy persons were indicated as 0 month after illness onset. Each dot represents an individual patient. Bars represent the median and interquartile range (IQR). The dotted line represents the threshold for positive in this assay. All serum samples were tested at least in duplicate. N, number; NA, not available; ns, not significant; **p < 0.01, ***p < 0.001.

We measured neutralizing antibodies in serum samples from confirmed COVID-19 cases at early and late time points after onset of illness. We found that 97.7% (42/43) of COVID-19 patients developed neutralizing antibodies with a median of 1:237 (IQR, 1:149–1:508) in 1- to 2.5-month sera, of which 69.0% (29/42) had a titer ≥1:128, and 21.4% (9/42) had a titer ≥1:512 (Figures 3A,B). Our data showed that most convalescent patients had detectable NAbs 10–11 months after illness onset and the seropositive rate remained at 91.9% (57/62) with the median of 1:99 (IQR, 1:47–1:212). However, 37.3% (19/51) of convalescent patients showed low levels of serum neutralizing antibodies between 1:8 and 1:64. Only a small proportion (5.9%) of patients had a serum neutralizing antibody titer ≥1:512. To explore how long neutralizing antibodies exist in convalescent patients, we tested neutralizing antibodies in the sera of these patients 17–18 months after illness onset. Surprisingly, we found that 88.9% (40/45) of patients had detectable NAbs 17–18 months after illness onset with a median of 1:80.0 (IQR, 1:40–1:164.5). Among them, 28.9% (13/45) showed low levels of serum neutralizing antibodies with NAbs between 1:8 and 1:64. No patient had a serum neutralizing antibody titer ≥1:512. The neutralizing antibody titers decreased from the early time point to the late time point after illness onset (1–2.5 months vs. 10–11 months, *p* < 0.001; 1–2.5 months vs. 17–18 months, *p* < 0.001). Besides, we observed a slight decline of the NAbs between the 10- to 11-month and the 17- to 18-month groups though the difference was not significant (*p* = 0.195) (Figures 3A,B).

Serum samples at different time points after illness onset showed differences in the distribution of NAbs (Figure 3B). The proportion of sera with a titer <1:64 increased from 1–2.5 months to 10–11 months (14.0–35.5%, *p* = 0.014) and remained stable from 10–11 to 17–18 months (35.4–40.0%, *p* = 0.006). The proportion with a titer >1:128 decreased from 1–2.5 to 10–11 months (76.7–38.7%, *p* < 0.001) and remained stable from 10–11 to 17–18 months (38.7–33.3%, *p* = 0.568). These data indicated that the neutralizing antibody declined from 1–2.5 to 10–11 months after illness onset, and remained relatively stable after that, even up to 17–18 months.

### Changes of SARS-CoV-2 Anti-S and Anti-N IgG

The IgG antibody positive rates to SARS-CoV-2 S protein were 95.2% (40/42), 85.5% (53/62), and 80.0% (36/45) with a medium OD (IQR) of 1.14 (0.72–1.75), 0.55 (0.29–0.86), and 0.53 (0.25–0.78) at the 1- to 2.5-month sera, 10- to 11-month sera, and 17- to 18-month sera, respectively. The IgG antibody positive rates to SARS-CoV-2 N protein were 97.6% (41/42), 82.3% (51/62), and 86.7% (39/45) with a medium OD (IQR) of 1.21 (0.47–2.07), 0.46 (0.28–1.15), and 0.66 (0.31–1.08) at the 1- to 2.5-month sera, 10- to 11-month sera, and 17- to 18-month sera, respectively. A progressive decline in IgG antibody to S protein (anti-S IgG) and IgG antibody to N protein (anti-N IgG) was observed from the 1st to the 18th month after illness onset (Figures 3C,D). We found that the anti-S IgG levels were strongly correlated with the anti-N IgG levels (Spearman rank correlation coefficients = 0.461, *p* < 0.001) (Figure 3E). The anti-S IgG and anti-N IgG levels were positively correlated with neutralizing antibody titers against SARS-CoV-2 in convalescent sera, respectively (Figures 3F,G).

### Comparison of Antibody Levels in Different Groups of Patients

**FIGURE 4:**
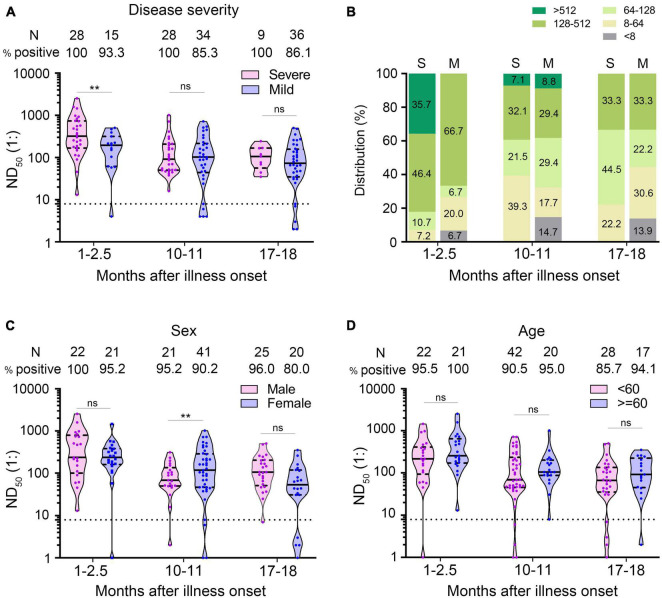
Comparison of neutralizing antibodies in different groups of patients. **(A)** ND_50_ of COVID-19 patients at different times after illness onset according to the severity of the disease. **(B)** Distribution of ND_50_ in sera of COVID-19 patients according to the severity of the disease. **(C,D)** The ND_50_ of COVID-19 patients according to sex **(C)** and age **(D)**. Bars represent the median and interquartile range (IQR). The dotted line represents the threshold for positive in this assay. All serum samples were tested at least in duplicate. Multivariate generalized linear models were used to compare the difference between ND_50_ and potential factors (i.e., disease severity, sex and age). **p < 0.01. ns, not significant; S, severe; M, mild; N, number.

**FIGURE 5:**
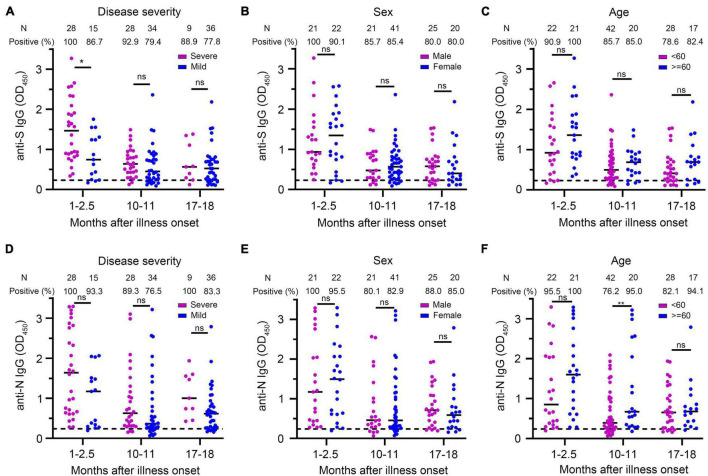
Comparison of anti-S IgG and anti-N IgG levels in different groups of patients. **(A–C)** Anti-S IgG levels of COVID-19 patients at different times after illness onset according to the severity of the disease **(A)**, sex **(B)**, and age **(C)**. **(D–F)** Anti-N IgG levels of COVID-19 patients at different times after illness onset according to the severity of the disease **(D)**, sex **(E)**, and age **(F)**. Bars represent the medium. Dotted lines represent the threshold for positive in these assays. Multivariate generalized linear models were used to compare the difference between antibody levels and potential factors (i.e., disease severity, sex, and age). *p < 0.05; **p < 0.01; ns, not significant; N, number.

We used multivariate GLM to estimate the association between neutralizing antibodies and potential factors (i.e., age, gender, and disease severity). It has been widely reported that the severity of the disease is a factor affecting the neutralizing antibody response (Choe et al., 2020; Lei et al., 2021; Yamayoshi et al., 2021). We explored whether the disease severity affected neutralizing antibody titer after a prolonged infection. Multivariate GLM analysis showed that the NAbs in the severe group were significantly higher than that in the mild group in the 1- to 2.5-month sera (β = −0.933, *p* = 0.004) (Figure 4A). We found that 35.7% (10/28) of severe patients developed high levels (>1:512) of neutralizing antibodies; in contrast, no mild patient maintained high titers in the 1- to 2.5-month sera (Figure 4B). Unexpectedly, 10–11 and 17–18 months after illness onset, the difference of the NAbs between the severe group and the mild group was not significantly different (severe group 105.9 vs. mild group 73.7 in 10–11 months, *p* = 0.946; severe group 95.4 vs. mild group 66.0 in 17–18 months, *p* = 0.636) (Figure 4A). There was no difference in the distribution of NAbs between the severe group and mild group of patients 10–11 or 17–18 months after COVID-19 onset (Figure 4B). Consistent with the trend of neutralizing antibodies, we found that anti-S IgG in the severe group was significantly higher than that in the mild group in the 1- to 2.5-month sera. The difference of anti-S IgG and anti-N IgG between the severe and mild group was not significant between the 10- to 11-month and 17- to 18-month sera (Figures 5A,D).

Multivariate GLM analysis showed that the NAb titers were not related to gender and age (Supplementary Table 1 and Figures 4C,D) and that anti-S IgG and anti-N IgG levels were not related to gender and age either (Supplementary Table 2 and Figures 5B,C,E,F).

### SARS-CoV-2-Specific T-Cell Memory Persisted at Least 17–18 Months After Illness Onset

**FIGURE 6:**
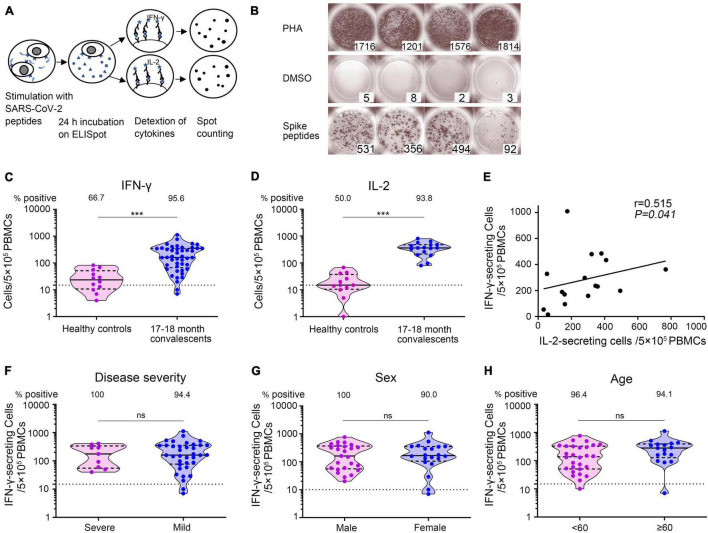
SARS-CoV-2-specific T-cell responses 17–18 months after COVID-19 onset. **(A)** Schematic of memory T-cell ELISpot assay. **(B)** Representative ELISpots from COVID-19 convalescent patients 17–18 months after illness onset with SARS-CoV-2 spike S1 and S2 mixture peptides as the specific stimulus, DMSO as the negative control, and PHA as the positive control. The numbers in the lower right corner represented the spots counting. **(C,D)** The total number of SARS-CoV-2-specific IFN-γ- secreting T cells and IL-2 secreting T cells per 5 × 10^5^ PBMCs. Each dot represents an individual patient. Bars represent the median and interquartile range (IQR). Statistical significance was determined using the Mann–Whitney *U*-test, and represented as ***p < 0.001. **(E)** Correlation of SARS-CoV-2-specific IFN-γ-secreting T cells and IL-2-secreting T cells 17–18 months after illness onset (*N* = 16). A two-sided Spearman rank correlation test was used to evaluate the correlation of the two cytokines. **(F–H)** IFN-γ-secreting T cells of COVID-19 patients according to disease severity **(F)**, sex **(G)**, and age **(H)**. Multivariate generalized linear models were used to compare the difference between IFN-γ spots and potential factors (i.e., disease severity, sex, and age). The dotted horizontal line represents the threshold for positive in this assay. ***p < 0.001. PBMCs, peripheral blood mononuclear cells; DMSO, dimethyl sulfoxide; PHA, phytohemagglutinin.

To determine whether SARS-CoV-2-specific T-cell immunity was generated and sustained in COVID-19 convalescent patients, we utilized ELISpot assay to measure the number of IFN-γ- and IL-2-secreting T cells, respectively (Figure 6A). PBMCs were obtained from 45 convalescent patients sampled 17–18 months after illness onset together with 12 unexposed and unvaccinated healthy individuals and then stimulated with SARS-CoV-2 spike S1 and S2 mixture peptides. Representative ELISpots from COVID-19 convalescent patients against SARS-CoV-2 antigens were presented, with PHA as positive and DMSO as negative control (Figure 6B). IFN-γ- as well as IL-2-secreting cell numbers were significantly higher in convalescent patients than in healthy persons. We found that 95.6% (43/45) of COVID-19 patients developed robust SARS-CoV-2-specific IFN-γ-secreting T-cell responses [median: 161 spot-forming cells (SFCs)/5 × 10^5^ PBMCs, IQR: 74, 351 SFCs/5 × 10^5^ PBMCs] and 93.8% (15/16) developed SARS-CoV-2-specific IL-2-secreting T-cell responses (median: 234 SFCs/5 × 10^5^ PBMCs, IQR: 167, 380 SFCs/5 × 10^5^ PBMCs) (Figures 6C,D). Patients with higher SARS-CoV-2-specific IFN-γ secreting T-cell numbers also had higher IL-2 secreting T-cell numbers (Spearman correlation coefficient = 0.515, *p* = 0.041) (Figure 6E). In addition, using multivariate GLM analysis, we found that there was no significant difference in the IFN-γ-secreting T-cell immune response against SARS-CoV-2 between the severe and mild groups (Supplementary Table 4 and Figure 6F), between the male and female groups (Supplementary Table 4 and Figure 6G), and between the younger (<60 years old) and older groups (≥60 years old) (Supplementary Table 4 and Figure 6H). These results demonstrated that SARS-CoV-2-specific T-cell responses persisted at least 17–18 months after illness onset.

### The SARS-CoV-2-Specific T-Cell Response Correlated With Neutralizing Antibody Level 17–18 Months After Infection

**FIGURE 7:**
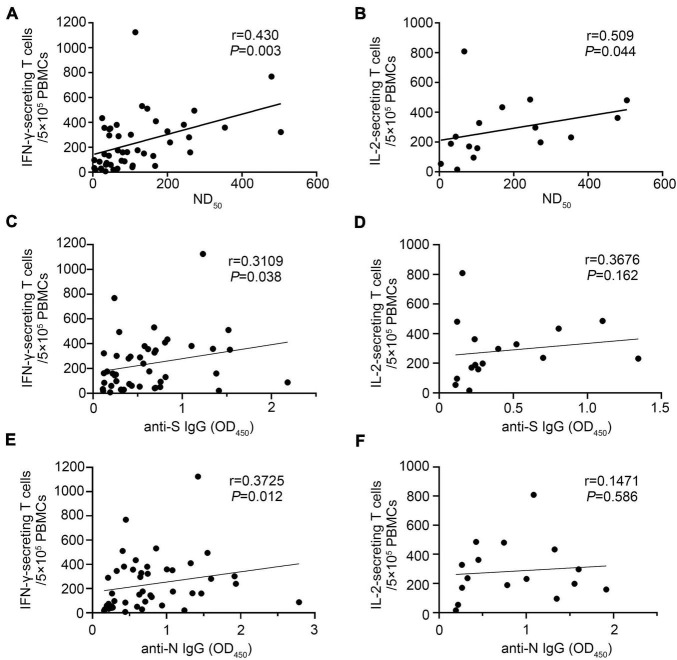
The SARS-CoV-2-specific T-cell response correlated with antibody levels 17–18 months after infection. IFN-γ- and IL-2-secreting T-cell responses and antibody (ND50, anti-S IgG, and anti-N IgG) levels were performed in COVID-19 convalescent patients 17–18 months after illness onset. **(A)** Correlation of ND_50_ and SARS-CoV-2-specific IFN-γ-secreting T cells (*N* = 45). **(B)** Correlation of ND_50_ and SARS-CoV-2-specific IL-2-secreting T cells (*N* = 16). **(C)** Correlation of anti-S IgG levels and SARS-CoV-2-specific IFN-γ-secreting T cells (*N* = 45). **(D)** Correlation of anti-S IgG levels and SARS-CoV-2-specific IL-2-secreting T cells (*N* = 16). **(E)** Correlation of anti-N IgG levels and SARS-CoV-2-specific IFN-γ-secreting T cells (*N* = 45). **(F)** Correlation of anti-N IgG levels and SARS-CoV-2-specific IL-2-secreting T cells (*N* = 16). A two-sided Spearman rank correlation test was used to evaluate the correlation between SARS-CoV-2-specific T-cell responses and antibody levels.

We found that IFN-γ-secreting T-cell numbers against SARS-CoV-2 strongly correlated with the titers of neutralizing antibody, and the Spearman rank correlation coefficient was 0.430 (*p* = 0.003) (Figure 7A). A similar correlation was observed between the IL-2-secreting T-cell numbers against SARS-CoV-2 and the titers of neutralizing antibody (*r* = 0.509, *p* = 0.044) (Figure 7B). We also found that SARS-CoV-2-specific IFN-γ-secreting T-cell responses were positively correlated with the anti-S and anti-N IgG levels, respectively (Figures 7C,E). Limited by the small sample size, we did not find that IL-2-secreting T-cell response was associated with the anti-S or anti-N IgG levels, respectively (Figures 7D,F). It is worth noting that five convalescent patients had no detectable neutralizing antibody response after 17- to 18-month infection, but four of them had a positive IFN-γ-secreting T-cell response.

## Discussion

The devastating COVID-19 outbreak is the biggest global health challenge in decades. Neutralizing antibodies and antigen-specific memory T cells had been implicated as critical for the control and elimination of viral infections. Previous studies have shown that neutralizing antibodies were significantly correlated with protection against SARS-CoV-2 infection in animal models (Deng et al., 2020; Hassan et al., 2020; Kim Y. I. et al., 2021; Selvaraj et al., 2021) and humans (Addetia et al., 2020; Huang et al., 2020; Legros et al., 2021). The presence of SARS-CoV-2-specific T-cell immunity was highly associated with protection from clinical disease after re-exposure in mice (Zhuang et al., 2021). Understanding the kinetics of neutralizing antibodies and cellular immunity to SARS-CoV-2 is critical for the prevention of reinfection.

Previous studies have reported that SARS-CoV-2 induced neutralization antibodies in the early time point of COVID-19 and neutralization antibodies declined with a prolonged recovery time from the disease (Choe et al., 2020; Ni et al., 2020; Wajnberg et al., 2020; Wang Y. et al., 2020; Zhou et al., 2020; Crawford et al., 2021). Several studies reported that antibodies against SARS-CoV-2 were detectable in most COVID-19 patients in a study period of up to 12 months (Choe et al., 2020; Dan et al., 2021; Feng et al., 2021; Peng et al., 2021; Wang H. et al., 2021; Zhang et al., 2021). The limitation of these studies is the short study period due to the recent emergence of SARS-CoV-2. We have no knowledge about the duration of neutralization antibodies in COVID-19 patients’ blood. Therefore, we carried out possibly the longest study period for SARS-CoV-2 neutralization antibody by recruiting patients from the first wave of COVID-19 in Wuhan City. We used HIV-1-based pseudovirus to evaluate the neutralizing antibody for SARS-CoV-2. Previous studies have shown that neutralizing antibody titers against SARS-CoV-2 pseudovirus correlated quite well with the results obtained using the live SARS-CoV-2 (Schmidt et al., 2020; Wang Y. et al., 2020; Sholukh et al., 2021; Wang H. et al., 2021; Yu et al., 2021). Due to not needing a biosafety laboratory and specificity of the SARS-CoV-2 pseudovirus, it has been widely used to detect neutralizing antibodies against SARS-CoV-2 since the COVID-19 outbreak (Robbiani et al., 2020; Tan et al., 2020; Wang Y. et al., 2020; Lei et al., 2021; Wang H. et al., 2021; Wang et al., 2021a,b; Yu et al., 2021).

We found that nearly all COVID-19 patients (97.7%) developed NAbs in the early time point within 2.5 months after illness onset and a majority of patients (88.9%) maintained NAbs for at least one and a half years. The neutralizing antibody titer waned slowly as the patient’s recovery time was extended. However, NAbs declined more rapidly within a year and significantly slowed afterward. It is possible that neutralizing antibodies induced by SARS-CoV-2 may persist for several years, which is supported by high positive rates of neutralizing antibodies against SARS-CoV-1 and MERS-CoV nearly 3 years after infection (Liu et al., 2006; Cao et al., 2007; Payne et al., 2016; Kim Y. S. et al., 2021). We found that anti-S IgG and anti-N IgG levels were strongly correlated with neutralizing antibody titers, indicating that IgG levels tested using ELISA could predict neutralizing antibody levels to some extent.

Several studies demonstrated that neutralizing antibody response was dependent on the severity of disease (Bošnjak et al., 2020; Choe et al., 2020). Consistent with previous studies at the early time of SARS-CoV-2 infection (Bošnjak et al., 2020; Choe et al., 2020; Long et al., 2020; Wang P. et al., 2020), we found that patients with severe infection tend to have a higher neutralizing antibody response than patients with mild infection. There is evidence that a high neutralizing antibody with severe SARS-CoV-2 infection was correlated with a higher viral load (To et al., 2020; Wang Y. et al., 2020). Surprisingly, the difference of the neutralizing antibody titers between the severe group and the mild group was not significant in 1 year to one and a half years after disease onset. It seems possible that neutralizing antibodies wane more dramatically in more severe patients than in mild patients, and this difference equalized after a long period of infection (Crawford et al., 2021). The same pattern was found in SARS-CoV-1. Cao et al. found that there were no significant differences in the specific antibodies against SARS-CoV-1 according to disease severity 30 months and even 36 months after infection (Cao et al., 2007).

The natural infection of COVID-19 elicits a massive T-cell immune response within a year (Grifoni et al., 2020; Ni et al., 2020; Zhou et al., 2020; Zhang et al., 2021). We found that a large majority of COVID-19 patients (more than 90%) developed SARS-CoV-2 S peptide-specific T-cell response in one and a half years post-infection, although T-cell responses vary greatly from person to person. From SARS, we learned that virus-specific memory T-cell response was detectable from 6 to 17 years after primary infection, suggesting a long-lasting immune memory (Tang et al., 2011; Ng et al., 2016; Le Bert et al., 2020). Our study provides evidence that COVID-19 infection could provide a lasting T-cell memory response.

Consistent with a previous study, a positive correlation was observed between SARS-CoV-2-specific T-cell responses and neutralizing antibody titers (Cassaniti et al., 2021), which suggested a correlation between cellular immunity and humoral responses. SARS-CoV-2 S specific T-cell responses were also found in 80% (4/5) of neutralizing antibody-negative COVID-19 patients, albeit at lower levels than in seropositive individuals. According to our data, the virus-specific T-cell response might be useful in cases of asymptomatic infection in the absence of a detectable antibody response against SARS-CoV-2.

Our data provide evidence that durable memory T-cell response was not significantly different between mild and severe patients one and a half years after SARS-CoV-2 infection. This suggested that the majority of mild and severe patients are likely to develop a long-lasting humoral and cellular immunity toward SARS-CoV-2, regardless of disease severity.

We found no significant differences in neutralizing antibody levels and T-cell responses between male and female individuals over time. Some studies reported that neutralizing antibody levels were higher in men than in women during the acute stage (Robbiani et al., 2020), which might be correlated with more severe symptoms and higher case fatality seen in men (Takahashi et al., 2020). Adjusting for disease severity and sex, we found that there was no significant difference in immunity level between those in the ≥60-year group and younger group. Neutralizing antibodies and virus-specific T-cell response could be detected in COVID-19 patients over 80 years old 17–18 months after disease onset. Thus, age was not considered to be a compromiser to antibody immune responses and SARS-CoV-2-specific T-cell responses (Lau et al., 2021). The effects of gender and age on the modulation of protective immunity needed to be explored in further studies.

Our study might be limited by the sample size and did not include asymptomatic patients. Due to limited information about the patients’ comorbidities in this study, we did not investigate the impact of diverse comorbidities on neutralizing antibody levels and T-cell response.

We concluded that SARS-CoV-2 infection induced robust and persistent neutralizing antibody response and SARS-CoV-2-specific T-cell responses at least one and a half years post-symptom onset in both mild and severe COVID-19 patients. Our findings may have crucial implications for COVID-19 epidemic control and long-term herd immunity.

## Data Availability Statement

The original contributions presented in the study are included in the article/Supplementary Material, further inquiries can be directed to the corresponding authors.

## Ethics Statement

The studies involving human participants were reviewed and approved by Wuhan University. The patients/participants provided their written informed consent to participate in this study.

## Author Contributions

X-JY: project conception. X-JY and L-NY: experimental design. L-NY, XX, P-PL, W-LL, S-HZ, X-GL, S-JZ, HL, Z-YW, FS, B-CL, YL, DL, and H-JH: sample collection. L-NY: experimental work and writing of original draft. L-NY and Z-DW: data analysis. X-JY, L-NY, and HY: revising and editing. All authors reviewed and approved the final the manuscript.

## Conflict of Interest

The authors declare that the research was conducted in the absence of any commercial or financial relationships that could be construed as a potential conflict of interest.

## Publisher’s Note

All claims expressed in this article are solely those of the authors and do not necessarily represent those of their affiliated organizations, or those of the publisher, the editors and the reviewers. Any product that may be evaluated in this article, or claim that may be made by its manufacturer, is not guaranteed or endorsed by the publisher.
